# Transcriptomic Changes and *satP* Gene Function Analysis in *Pasteurella multocida* with Different Levels of Resistance to Enrofloxacin

**DOI:** 10.3390/vetsci10040257

**Published:** 2023-03-28

**Authors:** Xue-Song Li, Yu Qi, Jun-Ze Xue, Guan-Yi Xu, Yu-Xuan Xu, Xuan-Yu Li, Inam Muhammad, Ling-Cong Kong, Hong-Xia Ma

**Affiliations:** 1 Department of Veterinary Medicine, College of Animal Science and Technology, Jilin Agricultural University, Xincheng Street No. 2888, Changchun 130118, China; 2 The Key Laboratory of New Veterinary Drug Research and Development of Jilin Province, Jilin Agricultural University, Xincheng Street No. 2888, Changchun 130118, China; 3 College of Life Sciences, Jilin Agricultural University, Xincheng Street No. 2888, Changchun 130118, China; 4 The Engineering Research Center of Bioreactor and Drug Development, Ministry of Education, Jilin Agricultural University, Xincheng Street No. 2888, Changchun 130118, China

**Keywords:** bovine respiratory disease, *Pasteurella multocida*, drug resistance, antibiotic tolerance, *satP*

## Abstract

**Simple Summary:**

*Pasteurella multocida* is a major pathogen of bovine respiratory disease, which is resistant to many of the commonly used antibiotics. We found that enrofloxacin has shown a high drug resistance in clinical treatment of *Pasteurella multocida* infection. In order to better understand the resistance mechanism of *Pasteurella multocida* to enrofloxacin, we isolated *PmS* and *PmR* strains with the same PFGE typing in vitro, and artificially induced *PmR* to obtain the highly resistant phenotype, *PmHR*. Then, transcriptome sequencing of clinically isolated sensitive strains, resistant and highly drug-resistant strains, treated with enrofloxacin at sub-inhibitory concentrations, were performed. The *satP* gene, of which the expression changed significantly with the increase in drug resistance, was screened. In order to further confirm the function of this gene, we constructed the deleted and complemented strains of *satP*, and further analyzed the function of the *satP* gene. After *satP* gene deletion, the resistance of *Pasteurella multocida* was obviously lower than that of wild-type strains in vitro, and the pathogenicity of *Pasteurella multocida* was reduced by about 400 times. We found that the *satP* gene is related to the tolerance and pathogenicity of *Pasteurella multocida*, and can be used as a target of enrofloxacin synergistic effect.

**Abstract:**

*Pasteurella multocida (Pm)* is one of the major pathogens of bovine respiratory disease (BRD), which can develop drug resistance to many of the commonly used antibiotics. Our earlier research group found that with clinical use of enrofloxacin, *Pm* was more likely to develop drug resistance to enrofloxacin. In order to better understand the resistance mechanism of *Pm* to enrofloxacin, we isolated *PmS* and *PmR* strains with the same PFGE typing in vitro, and artificially induced *PmR* to obtain the highly resistant phenotype, *PmHR*. Then transcriptome sequencing of clinically isolated sensitive strains, resistant and highly drug-resistant strains, treated with enrofloxacin at sub-inhibitory concentrations, were performed. The *satP* gene, of which the expression changed significantly with the increase in drug resistance, was screened. In order to further confirm the function of this gene, we constructed a *satP* deletion (*ΔPm*) strain using suicide vector plasmid pRE112, and constructed the *C-Pm* strain using pBBR1-MCS, and further analyzed the function of the *satP* gene. Through a continuously induced resistance test, it was found that the resistance rate of *ΔPm* was obviously lower than that of *Pm* in vitro. MDK_99_, agar diffusion and mutation frequency experiments showed significantly lower tolerance of *ΔPm* than the wild-type strains. The pathogenicity of *ΔPm* and *Pm* was measured by an acute pathogenicity test in mice, and it was found that the pathogenicity of *ΔPm* was reduced by about 400 times. Therefore, this study found that the *satP* gene was related to the tolerance and pathogenicity of *Pm*, and may be used as a target of enrofloxacin synergistic effect.

## 1. Introduction

*Pasteurella multocida (Pm)* is a Gram-negative bacterium implicated in multi-host infections. Strains of their species are currently classified into type A, type B, type D, type E and type F serogroups [1,2]. *Pm* is one of the primary pathogens of bovine respiratory disease (BRD) in Asia, Middle East, Africa and Europe [3,4,5]. At present, the infection of cattle with *Pm* leads to tremendous economic losses for the cattle industry due to increased mortalities and high treatment costs [6]. The treatment of BRD mainly depends on antibiotics, and penicillin has often been used as the drug of choice for the last few decades [7,8]. However, due to the irrational use of antibiotics in clinical treatment, it has been observed that the drug resistance rate of β-lactams, macrolides and tetracyclines has increased in recent years [9,10,11,12]. Irrational use of antibiotics to prevent the threat of BRD increases the rate of *Pm* resistance [13].

Fluoroquinolones are one of the main therapeutic drugs for *Pm* infections in animals [12]. Enrofloxacin has a good therapeutic effect on nasal, upper respiratory and lung infections caused by *Pm* infection in animals [14,15,16]. Unfortunately, *Pm* has recently been reported to possess acquired fluoroquinolone resistance mechanisms [17]. Among them, variation of DNA gyrase or topoisomerase IV, bacterial cell membrane permeability and bacterial active efflux mechanisms are the most common. DNA gyrase is a tetramer composed of GyrA and GyrB proteins, and topoisomerase VI is a tetramer composed of ParC and ParE proteins [18]. Mutation of any of these four proteins may cause drug resistance to fluoroquinolones. In *Pm*, *gyrA* gene mutation often leads to the structural change of DNA helicase, which leads to drug resistance. Within the *gyrA* gene, quinolone-resistant determining region (QRDR) is closely associated with drug resistance [19]. Mutation in this region can cause drug resistance. The specific positions of proteins encoded by this region are different across different strains. For example, they correspond to Gly–Asp94 in *Mycobacterium tuberculosis*, Ala67–Gln106 in *Escherichia coli* and Arg88Ile in *Pm*. Detection of QRDR gene mutation has been a hot issue in the early study of fluoroquinolone resistance [20]. Our previous results indicated that *Pm* is extremely resistant to fluoroquinolones in vivo and in vitro, and the mechanism is mainly mediated through target mutation in *gyrA* and *parC* [21]. The variation of DNA gyrase or topoisomerase VI is the main cause of bacterial resistance to fluoroquinolones. 

The bacterial cell membrane is a highly selective permeable barrier. There are some special proteins in the outer membrane of bacteria, which are called outer membrane proteins (OMPs). OMPs account for about half of the cell wall in Gram-negative bacteria. The OMPs have the functions of antibiotic, iron transport, host adhesion and maintenance of membrane integrity [22]. At present, it is clear that OmpF, TolC and OmpX are related to fluoroquinolone resistance [23]. Simultaneously, there is an energy-dependent protein efflux pump in the inner membrane of *Pm* which can discharge drugs, resulting in drug resistance [24]. The efflux of fluoroquinolones in *Pm* is mainly related to the survival–nodulation–cell division (RND) family, which includes the transporters of calcium, cobalt and nickel [25]. Among Gram-negative bacteria, the AcrAB–TolC system is the main active efflux pump related to fluoroquinolones. The active efflux pump and the change of bacterial outer membrane permeability play an important role in multidrug resistance, and attention has been paid to this by researchers [26]. 

However, the development of antimicrobial resistance is a complicated multifactorial process, and related studies have shown that the formation of drug resistance also depends on tolerance factors [27,28]. Antibiotic tolerance is the ability of antibiotic-sensitive bacteria at the gene level to survive under the influence of antibiotic treatment concentration, which plays a key role in the process of bacterial infection. Antibiotic tolerance is involved in chronic and recurrent infections [29]. Thus, some genes, such as *recA*, are essential for increased tolerance to antibiotic treatment by enhancing repair of DNA damage that occurs directly from antibiotic-induced DNA damage. Relevant studies have confirmed that amino acid mutations are related to the generation of tolerance [30,31]. Frequent use of antibiotics can enhance the antibiotic resistance of *Pm* [32]. Due to the importance of tolerance, there is a crucial need to identify new drug targets. Mechanistically, *Pm* tolerance to fluoroquinolones and the main targets for mediating tolerance have not been reported. To determine whether the resistance mechanism of *Pm* is caused by tolerance, it is crucial to work on a new tolerance mechanism and identify targets that can inhibit drug resistance.

## 2. Materials and Methods

### 2.1. Antibiotics, Culture Media, Bacterial Strains and Growth Conditions

The clinical *PmS* (*Pasteurella multocida* sensitive to ENR) and *PmR* (*Pasteurella multocida* resistant to ENR) were isolated from the lungs of dead cattle that have suffered from BRD in Shuangyang City, Jilin Province. The highly resistant strain (*PmHR* MIC of ENR is 64 μg/mL) was obtained according to the methods reported in previous studies [21]. The *PmS*, *PmR* and *PmHR* had the same molecular type after PFGE typing (previous research of our group) [8]. *Pm* was cultured on a brain–heart infusion (BHI) medium. Commercial antibiotics were purchased from Sigma-Aldrich Company. According to CLSI regulations, 20,480 μg/mL storage solution was prepared and frozen at −20 °C for later use. Growth was monitored by measuring the OD_600_ of the liquid cultures using a Biophotometer (Nanodrop2000, Thermo, Thermo Fisher Scientific, Waltham, MA, USA).

### 2.2. Determination of the MIC of Fluoroquinolones

The minimum inhibitory concentration (MIC) was established by broth microdilution and two-fold agar dilution, as recommended by the Clinical and Laboratory Standard Institute guidelines in VET01-A4. The MICs of ciprofloxacin and enrofloxacin were tested in the range of 0.03 to 512 µg/mL. The *Pm* cells were inoculated into BHI media and incubated for 24h at 37 °C. Then, cells were harvested and washed three times with PBS. Each strain was inoculated at a density of 10^5^ CFU/mL in BHI containing 0.03 to 512 µg/mL of ciprofloxacin and enrofloxacin, and grown for 24 h at 37 °C. The breakpoints used for fluoroquinolone were adopted from the CLSI document, VET01-A4. *Escherichia coli* ATCC25922 was used as a quality control strain.

### 2.3. Amplification and Sequencing of the QRDR Genes

Fluoroquinolone QRDR targets, *gyrA*, *gyrB*, *parC* and *parE* genes, were amplified by polymerase chain reaction (PCR) and compared using the *gyrA*-F (5’-CCTTATCGTAAATCCGCTCGTA-3’), *gyrA*-R (5’-CGCAGGGACTTTGGTTGG-3’), *gyrB*-F (5’-GAAATGACCCGCCGTAA-3’), *gyrB*-R (5’-CTTGCCTTTCTTCACTTTGTA-3’), *parC*-F (5’-TACGAAGGCATTGAACAAAC-3’), *parC*-R (5’-CCACTGTCCCTTGCCCTA-3’), *parE*-F (5’-AATACGGTAAAGCGGTGGC-3’) and *parE*-R (5’-GGATTCTGCTGGCGGTTC-3’) primers [10]. Amplified PCR products (312 bp, 456 bp, 421 bp and 331 bp) were cloned into the pMD18-T (from TaKaRa, TaKaRa Biotechnology (Dalian) Co., Ltd., Dalian, China) easy vector via TA cloning and sequenced for identification of mutation positions.

### 2.4. RNA Extraction, Library Construction, and Sequencing

The cells were grown to an exponential phase (OD_600_ = 0.5) and treated with ENR (sub-inhibitory concentration) over a period of 15 min. Total RNA extraction of *PmS*, *PmR* and *PmHR* was performed using an RNAiso Pure RNA Isolation Kit (TaKaRa, TaKaRa Biotechnology (Dalian) Co., Ltd., China), according to the manufacturer’s instruction. The quality and quantity of RNA was assessed, and the library construction and sequencing were performed at Biomarker Technology (Beijing, China) using the Illumina HiSeq 2500 platform. The genome sequence and annotation information of *Pm* were obtained from the NCBI database (Accession No. NC_014259.1). Quality-filtered reads were aligned to the reference genome sequence, using the CLC Genomics Workbench 4.0 (CLC Bio, Qiagen, Dusseldorf, *GER*). 

To identify DEGs (differential expression genes) among the three different samples, the expression level for each transcript was calculated using the fragments of RPKM (read per kilo base per million mapped reads) method. edgeR (https://bioconductor.org/packages/release/bioc/html/edgeR.html, accessed on 11 November 2021) was used for differential expression analysis. The DEGs between the two samples were selected using the following criteria: (i) the logarithmic of fold-change should be greater than 2 and the false discovery rate (FDR) should be less than 0.05. To understand the functions of the differentially expressed gene, GO functional enrichment and KEGG pathway analyses were carried out by Goatools (https://github.com/tanghaibao/Goatools, accessed on 27 November 2021) and KOBAS (http://kobas.cbi.pku.edu.cn/home.do, accessed on 27 November 2021), respectively. DEGs were significantly enriched in terms of GO and metabolic pathways when their Bonferroni-corrected p-value was less than 0.05. Then, with the increase in drug resistance, differentially expressed genes were screened. 

### 2.5. Quantitative Reverse Transcription PCR (RT-qPCR) Analysis

Expression of *satP*, *focA*, *napF*, *glgP* and *napD* genes was evaluated by qPCR at the mRNA level. The cells were grown to an exponential phase (OD_600_ = 0.5) and treated with ENR (sub-inhibitory concentration) over a period of 15 min. Total RNA extraction of *PmS*, *PmR* and *PmHR* was performed using an RNAiso Pure RNA Isolation Kit (TaKaRa), according to the manufacturer’s instruction, and was reverse-transcribed to cDNA. The real-time PCR process was performed on the Applied Biosystems 7500 Real-Time PCR System (ABI, state abbr., USA). The primers of *satP*-F (CGCCAAAAGCAAACGCCATACC) and *satP*-R (TACGCCAATCCGGGTCCATTAGG) were designed. The specificity of the qRT-PCR results was confirmed by agarose gel and melting curve analysis, and the data were analyzed by the Applied Biosystems™ 7500 software. All qPCR reactions were performed with six replicates.

### 2.6. Construction satP of the Deletion Strains and the Complemented Strains 

The *satP* gene knockout was conducted with the suicide vector, pRE112. Briefly, the genome of the strain to be knocked out was amplified by PCR using UP-F/R and DOWN-F/R primers (Supplementary Table S1), respectively, to obtain the upstream and downstream regions of the *satP* gene. Then, the PCR products were mixed as the template for the next overlapping PCR, and UP-F and DOWN-R were used as primers to amplify the template. Finally, the amplified PCR product (1673 bp) was cloned into the PRE112 vector, and no base mutation was identified by sequencing. Then, the plasmid pRE112-*satP* was successively transformed into DH5α-λpir and *E. coli* WM3064. The single colony was inoculated into 5 mL BHI broth, then inoculated into the BHI agar medium containing 10% sucrose, and cultured overnight at 37 °C. The recombinant strain without the *satP* gene and pRE112 plasmid was screened (thus creating the *satP* mutant of the *ΔPmS*, *ΔPmR* and *ΔPmHR* deletion strains). The complemented strain, *CPmS*, was constructed with the expression plasmids pBBR1-MCS, by homologous recombination.

### 2.7. Tolerance of Wild-Type and satP Mutant 

Tolerance was determined according to Westfall’s and Cirz’s method [33,34]. The tolerance of enrofloxacin was detected by micro broth dilution method and (MDK_99_) test (the minimum duration for killing 99% of cells), respectively. In short, wild-type strains, deletion strains and complemented strains were cultured to OD_600_ = 0.5 and colonies were counted. The bacterial solution was diluted to a final concentration of 10^6^ CFU/mL according to the counting results. The final concentration of ENR with MIC was added to the culture medium, 50 μL per hour was taken, and the colonies were counted after dilution. The line diagram of MDK_99_ was made with the number of colonies as abscissa and time as ordinate. The experiment was set up three times. At the same time, 200 μL per hour was added into a 96-well plate, diluted 10 times continuously, and participated in dilution 5 times. The diluted bacterial solution was inoculated on the BHI solid plate through a multi-point inoculator. The growth of the colonies was observed the next day and the results recorded. 

### 2.8. Animal Studies

Female BALB/c mice, aged 6–8 weeks (18–20 g), were obtained from the Animal Experimental Center of Jilin University (Changchun, China). All experiments were carried out under the Regulations of Jilin Laboratory Animal Management Committee of Jilin Province. The license number of experimental animals was JLAU20210423001, which is certified by Jilin Science and Technology Association.

#### 2.8.1. Pathogenicity Test in Mice 

Twenty-five mice, both male and female, were randomly divided into five groups, with five mice in each group. After 3 days of adaptive feeding, the mice were reared without food and water for 12 h, and then *PmS* with bacterial counts of 10^8^, 10^7^, 10^6^, 10^5^ and 10^4^ CFU/mL were intraperitoneally injected in mice. The condition of the mice was observed within seven days and the doses leading to the death of the mice were recorded. The LD_50_ of the *PmS* strain was calculated by the Bliss method. The LD_50_ of the *ΔPmS* and *CPmS* strains was determined by the same method as that of the *PmS* strain. 

Fifty mice, half male and half female, were randomly divided into five groups, with 10 mice in each group. After adaptive feeding for 3 days, the mice were reared without food and water for 12 h. According to the results of the preliminary experiment, each strain of bovine type A *Pm* was diluted to different concentrations, and each strain was set with five concentration gradients. The infection was carried out by intraperitoneal injection, and each mouse was infected with 100 μL. The control group was injected with the same dose of sterile PBS buffer. After 48 h of observation, the death rate was noted. The LD_50_ of each strain in the mice was calculated by the Bliss method, using the SPSS 26.0 software.

#### 2.8.2. Acute Pathogenicity Protection Test in Mice

Sixty mice, half male and half female, were randomly divided into six groups, with 10 mice in each group. After 3 days of adaptive feeding, the mice were kept without food and water for 12 h. The bacterial solution was centrifuged to remove the culture medium, then resuspended with normal saline, and the mice were injected with twice the concentration of LD_50_. The first and second groups were injected with *PmS* bacterial solution, the third and fourth groups were injected with *ΔPmS* bacterial solution, and the fifth and sixth groups were injected with the *CPmS* bacterial solution.

One hour after bacterial injection, mice in the first, third and fifth groups were intramuscularly injected with a 100 μL of ENR (2.5 mg/kg body weight) twice a day. The second and the fourth groups were injected with the same dose of normal saline intramuscular, and the survival conditions of the mice in each group were observed and recorded, and plotted by the Prism software, according to the previously reported methods [34,35].

## 3. Results

### 3.1. Selection of Fluoroquinolone-Resistant Mutants

Two strains (*PmS* and *PmR*) with the same molecular typing and different fluoroquinolone resistance phenotypes were isolated by 16S rRNA and PFGE (Supplementary Figure S1). A highly drug-resistant strain, *PmHR*, was obtained by in vitro fluoroquinolone induction, using *PmS* as the parental strain. By detecting the QRDR region, we found that *PmS* had no QRDR mutation, and *PmR* and *PmHR* had three identical QRDR mutations. (Table 1). It was noteworthy that the mutations in the *gyrA*, *gyrB* and *parC* genes of the highly resistant strain, *PmHR*, were the same as those of the resistant strain, *PmR*, which indicates that the difference in enrofloxacin resistance level was not due to QRDR mutations, and may have other forms of drug resistance mechanism.

The parent strain and mutant-derived strains were tested for carbonyl cyanide 3-chlorophenylhydrazone (CCCP)- and verapamil-sensitive efflux. The MICs of ENR were determined using the agar dilution method in the presence and absence of CCCP (4 µg/mL, MIC/2) and verapamil (8 µg/mL, MIC/2). These results indicated that the efflux pump genes did not play any role in the quinolone resistance of *Pm* mutants selected in vitro. The characteristic features of the three lineages are described in Table 1. We speculated that *PmHR* might be tolerant to ENR, and tolerance mechanisms should be investigated in future studies.

### 3.2. Transcriptional Profiling of PmS, PmR and PmHR by RNA Sequencing

In order to search for genes that played an important role in the formation of fluoroquinolone resistance in *Pm*, we sequenced the transcriptome of three strains under the sub-inhibitory concentration of ENR (Supplementary Table S2). The results showed that more than 97% of the reads can be mapped to the reference genome sequence (GenBank: 8172276). Raw read data were sequenced and filtered for further analysis, yielding approximately 1.76–2.15 billion clean reads. The average length of the reads for *PmS*, *PmR* and *PmHR* was 300bp, and their GC content was 42.77%, 43.17% and 42.35%, respectively, indicating successful sequencing of the *Pm* with different levels of ENR resistance (Supplementary Table S3). 

In total, 14,055,756 clean reads were detected. Based on the FDR threshold ≤0.05 and |log2 fold-change (FC)| > 1, a total of 1,499 significant DEGs were identified. Simply put, compared with *PmS*, *PmR* has 144 up-regulated and 292 down-regulated genes. Compared with *PmS*, 181 genes were up-regulated and 382 genes were down-regulated in *PmHR*. Compared with *PmR*, 104 genes were up-regulated and 172 genes were down-regulated in *PmHR* (Supplementary Figures S2 and S3). The GO enrichment analysis and KEGG pathways were performed for DEGs. Enrichment analysis revealed significant changes in 19 GO biology processes, among which, seven biology processes were relating biological process, seven biology processes were relating cellular components and five biology processes were related to molecular function. These processes involve the intrinsic components and compositions of cell membranes, intracellular organelles, adhesion proteins and membrane-protein-related pathways. (Supplementary Figure S4). On the other hand, the top 20 of KEGG pathways were annotated. We annotated 650 DEGs and enriched them into 215 metabolic pathways. We further screened the enriched metabolic pathways, and categorized them into metabolic pathways such as antigen presentation and processing, cell adhesion, phagocytosis and cell killing and virulence. Among them, we found that antigen presentation and processing enriched DEGs were the most common. (Supplementary Figure S5 and Table S4). 

**Figure 1:**
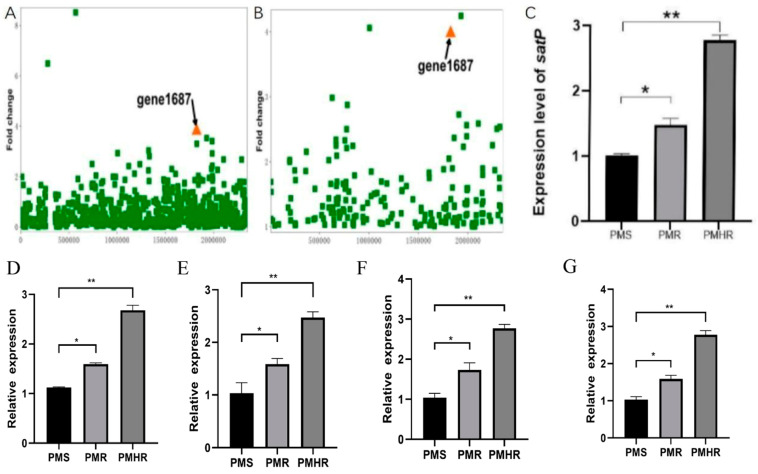
Characterization and verification of differentially expressed genes. (**A**) Differentially expressed gene analysis of resistance groups (*PmS* vs. *PmR*). (**B**) Differentially expressed gene analysis of resistance groups (*PmS* vs. *PmHR*). (**C**) *satP*, (**D**) *focA*, (**E**) *napF*, (**F**) *glgP* and (**G**) *napD* gene expression level verification using qRT-PCR. (All data are expressed as the mean ± SD. *n* = 3. ∗∗ *p* < 0.01 vs., ∗ *p* < 0.05 vs. control group).

Furthermore, in order to find the difference between *PmR* and *PmHR*, significantly expressed genes were screened with the improvement of drug resistance, and the *satP* (gene 1687), *focA*, *napF*, *glgP* and *napD* genes were significantly up-regulated (Table S5). As the most significantly expressed gene, the *satP* gene is mainly involved in encoding intimal proteins. By analyzing the expression of *satP*, we found that the expression of *satP* in the *PmR* group was 17 times higher than that in the *PmS* group, and the expression of *satP* in the *PmHR* group was 20 times higher than that in the *PmS* group (Figure 1A,B).

The qRT-PCR analysis was performed to validate the expression of *satP*, *focA*, *napF*, *glgP* and *napD* in *Pm* with different levels of drug resistance. As shown in Figure 1C–G, qRT-PCR results of *satP* agreed with the FPKM data. At the same time, the relative expression of *satP* in the *PmR* group was significantly higher than in the *PmS* group, and in the *PmHR* group significantly higher than in the *PmR* group. The expression level of *satP* gene was in accordance with the trend of drug resistance.

### 3.3. Construction and Detection of satP Strains

**Figure 2:**
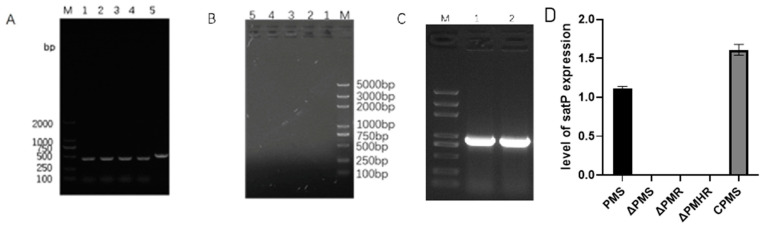
(**A**) Confirmation that the deletion of the *satP* strains were genetically stable beyond 40 generations by PCR. M: DL 2000 Marker; 1–4, deletions strain *satP*; 5, wild-type strain. (**B**) The genetic stability of *satP*-deficient strains over 40 generations was verified by pRE112 primers. M: 2000 plus DNA Marker; 1–4, deletions strain *satP*; 5, wild-type strain. (**C**) Confirmation that the *CPmS* was genetically stable beyond 40 generations by PCR. M, 2K plus DNA Marker; 1, wild-type strain; 2, *CP*m. (All data are expressed as the mean ± SD. *n* = 3). (**D**) The expression level of the *satP* gene in the wild-type strain, deletion strains and *CPmS*.

The knockout of *satP* in *PmS*, *PmR* and *PmHR* was conducted with the suicide vector pRE112. To determine whether *ΔPmS*, *ΔPmR*, *ΔPmHR* and *CPmS* were successfully constructed, hereditary stability, PCR detection and qRT-PCR were performed. PCR showed that the *satP* deletion strains were 700bp less than that of the wild-type strain and *CPmS*. Real-time qRT-PCR showed that no signal of the *satP* gene was detected from the deletion strains. Genetic stability showed that *ΔPmS*, *ΔPmR*, *ΔPmHR* and *CPmS* were successfully constructed. The deletion of *satP* did not affect the MIC and growth of the strains (Figure 2A–D). 

We found that when the *satP* gene was deleted, *ΔPmS*, *ΔPmR* and *ΔPmHR* had no QRDR mutation compared with those without deletion (Table 2). The MIC of ENR was determined by the agar dilution method in the presence and absence of CCCP (4 g/mL, MIC/2) and verapamil (8 g/mL, MIC/2). The results showed that the function of the efflux pump had no effect on the phenotype of *Pm* to ENR. 

### 3.4. Effect of satP Deletion on Drug Resistance Formation

**Figure 3:**
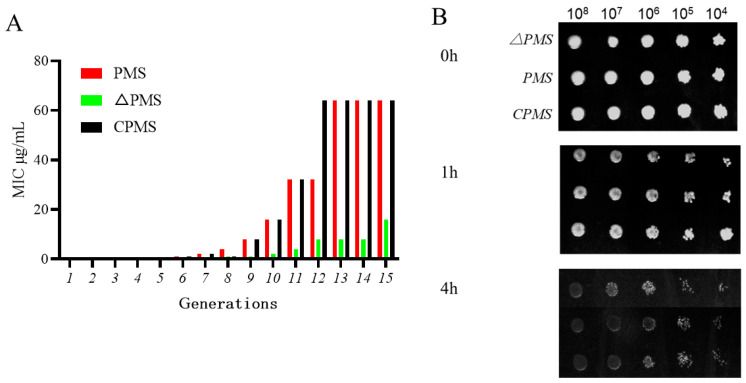
Effect of *satP* deletion on drug resistance formation and tolerance formation. (**A**) Development time of fluoroquinolone resistance of *PmS*, *ΔPmS* and *CPmS*, induced by sub-inhibitory concentrations of enrofloxacin. (**B**) Tolerance test of *ΔPmS* to enrofloxacin microdilution method.

To verify the effect of the *satP* gene on ENR resistance of *Pm*, an in vitro method of inducing drug resistance was used to detect the development time of drug resistance of the gene deletion strain, wild-type strain and replenishment strain. The results showed that in generation 1–5, the drug resistance of *PmS* was the same as that of the *ΔPmS* and the *CPmS*, with an MIC of 0.5 μg/mL. The drug resistance formation time of sensitive strains and gene deletion strains was different in generation 5–10. The resistance of *PmS* and the *CPmS* developed rapidly, with an MIC of 64 μg/mL in the 10th generation. While the drug resistance of *ΔPmS* developed slowly, and the MIC was 16 μg/mL in the 10th generation (Figure 3A).

### 3.5. Effect of ΔPmS on Drug Tolerance Formation

The results showed that in 10×MIC enrofloxacin treatment, the killing effect of the *ΔPmS* was faster than the *PmS* and *CPmS*, and the tolerance of the *satP* gene deletion strains to ENR was obviously weakened. The results of mutation frequency showed that the mutation frequency of the deletion strains decreased significantly (Figure 3B and Table 3). 

**Figure 4:**
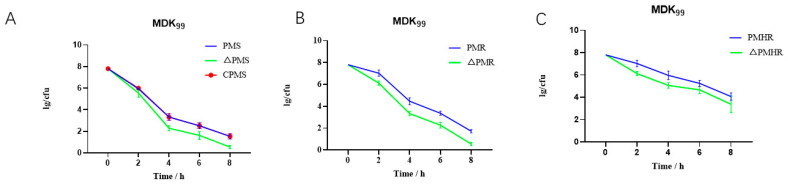
The expression level of the *satP* gene in the wild-type S, deletion *satP*. MDK_99_ and survival proportions. (**A**–**C**) MDK_99_ of *PmS*, *PmR*, *PmHR*, *ΔPmS*, *ΔPmR* and *ΔPmHR*.

In order to further verify the effect of the *satP* gene deletion on *Pm* tolerance, MDK_99_ was measured. The result showed that the speed of killing of *ΔPmS*, *ΔPmR* and *ΔPmHR* was faster than that of *PmS*, *PmR* and *PmHR*, which reached 10^5^ CFU/mL. The results showed that the deletion of the *satP* gene could lead to the decrease in tolerance (Figure 4A–C).

### 3.6. Determination of Median Lethal Dose

The amount of bacteria and survival rate of 40 mice, injected intraperitoneally, were shown in Supplementary Table S6. The reasonable detection ranges were 10^5^–10^7^ CFU/mL and 10^9^–10^11^ CFU/mL. According to the data in Table S6, the LD_50_ of *PmS* was 1.8 × 10^6^ CFU/mL, and the 95% confidence limit was 1.3 × 10^6^–2.5 × 10^6^ CFU/mL, the LD_50_ of *ΔPmS* was 7.1 × 10^9^ CFU/mL and the 95% confidence limit was 5.3 × 10^9^–9.4 × 10^9^ CFU/mL, the LD_50_ of *CPmS* was 1.8 × 10^6^ CFU/mL and the 95% confidence limit was 1.3 × 10^6^–2.5 × 10^6^ CFU/mL.

### 3.7. Acute Pathogenicity Protection Test in Mice

**Figure 5:**
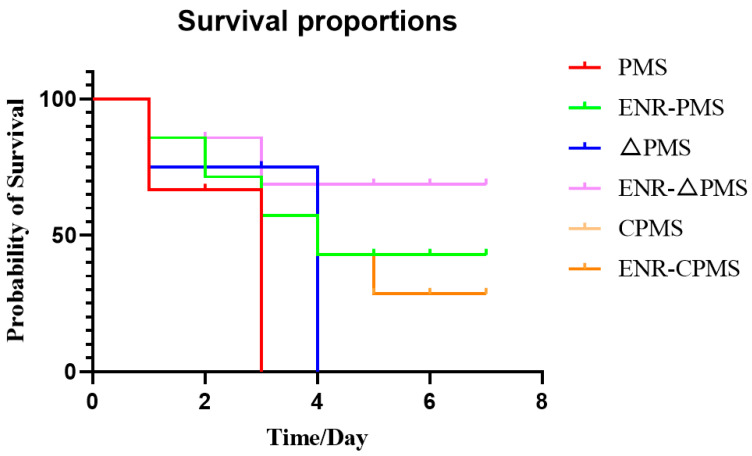
Survival rates of the mice treated with ENR infected by *PmS* and *ΔPmS*.

In order to evaluate the effect of the *satP* gene deletion on the infection model, we first constructed the infection model of *PmS*, *CPmS* and *ΔPmS* in mice, and then injected ENR intramuscularly with a clinical therapeutic within the experimental group, and injected normal saline intramuscularly within the control group. The results are shown in Figure 5. Compared with *PmS*, *ΔPmS* was more likely to be treated with ENR.

## 4. Discussion

BRD has become one of the major diseases in the global cattle industry [36]. One of the main pathogens causing this disease is *Pm*. At present, the treatment of *Pm* mainly depends on antibiotics. However, due to the irrational use of antibiotics in clinical treatment, *Pm* is prone to drug resistance. In China, fluoroquinolones are commonly used to treat BRD infected by *Pm* [37]. In recent years, multidrug-resistant strains of *Pm* have appeared, and *Pm* has caused huge economic losses to the cattle industry worldwide. Tang *et al.* discovered that 93.2% of the 92 *Pm* strains were resistant to fluoroquinolones [36]. The mutation of the drug resistance target gene has a vital impact on the formation of drug resistance. Kong et al. detected the target genes in quinolone-resistant *Pm* and found that the mutation of *gyr*A and *par*C genes had a significant influence on the generation of drug resistance [37]. 

In this study, three strains (*PmS*, *PmR* and *PmHR*) with different resistance phenotypes in relation to fluoroquinolones were screened. We found that when the *satP* gene was deleted, *ΔPmS*, *ΔPmR* and *ΔPmHR* had no QRDR mutation, compared with those without deletion. The different levels of resistance between *PmHR* and *PmR* might be mediated by other resistance mechanisms or tolerance. Relevant research has shown that antibiotic tolerance can increase the probability of antibiotic resistance [38]. With respect to the research question, we sequenced the transcriptome of three strains of bovine capsular *Pm* with different levels of drug resistance, treated by sub-inhibitory concentration, and counted the differential genes. On the basis of ensuring stable and accurate transcriptome data, we analyzed the functional annotation and metabolic pathways, and analyzed and screened the genes that might be related to the formation of drug resistance. The results showed that with the increase in drug resistance, the expression of some genes, namely *satP*, *focA*, *napF*, *glgP* and *napD*, increased significantly. The most significant difference was the *satP* gene, which was up-regulated 17 times in the drug-resistant group and 20 times in the highly drug-resistant group compared with the sensitive group. *satP* is a representative member of the AceTr family, which is preliminarily identified as a succinate acetate/proton cotransporter transmembrane protein [39]. Indrajiths et al. found that the outer membrane protein, ompk36, is an important influencing factor of cefoxitin and carbapenem resistance [40]. Yamamoto et al. found that the membrane protein mmpl5 has an effect on the resistance of clofazimine and is also related to the tolerance of a variety of antibiotics [41]. Lu et al. studied the function of membrane channel protein, *lamB*, and found that it can produce antibiotic resistance by reducing the pressure of protein translation and DNA replication. In order to further detect antibiotic resistance, the *lamB* gene was knocked out and was found to increase antibiotic resistance by reducing the activity of metabolic pathways such as the tricarboxylic acid (TCA) cycle, pentose phosphate pathway and glycolysis/gluconeogenesis. By comparing the function of the wild-type strain and deletion strain after ciprofloxacin treatment, it was found that the down-regulation of the *lamB* gene can increase the resistance of bacteria to antibiotics [42].

Based on the research background of the *satP* gene, in order to further confirm the function of the *satP* gene, we constructed the gene deletion strain of a sensitive strain and drug-resistant strain. The results showed that the tolerance of *satP* decreased after the deletion of the *satP* gene. Safi et al. have pointed out that when antibiotics act on bacteria, antibiotic tolerance first appears. When antibiotics continue to act, antibiotic tolerance will aggravate the generation of drug resistance. Compared with the life cycle of drug resistance phenotype, antibiotic tolerance is a temporary state. Timely release of antibiotics can restore its tolerance, so the tolerance is reversible [43]. Repeated exposure will maintain the tolerance [44], which reaffirms the foundation for functional studies. During the research of this gene in *Pm*, Westfall et al. studied the mechanism of *MRSA* tolerance by using triclosan, an antibacterial agent synthesized by fatty acids, to induce the change of *MRSA* tolerance. MDK_99_ and the agar diffusion method were used to detect its tolerance. The results showed that triclosan increased the tolerance of *LL* to bactericidal antibiotics by 10,000 times, and reduced the efficacy of antibiotics by 100 times. Genetic analysis showed that the antibiotic tolerance mediated by triclosan needs the cooperation of ppGpp [33]. However, until now, there was no report about *satP*-gene-mediated tolerance; other membrane transporters have been confirmed to be involved in the formation of tolerance. Poudyal et al. found a *MerR*-type transcriptional activator, which can activate the *BrlR* target encoding ABC transport system gene and affect the drug tolerance of bacteria [45]. One of the main stress responses of almost all bacteria depends on the rapid accumulation of ppGpp, an alarmone. Many scholars have found that ppGpp has a great impact on bacterial tolerance. Recetntly, Ronneau et al. and others have studied ppGpp and found that the concentration of ppGpp is determined by a highly conserved and widely distributed protein family, and the family is known as Rela-Spot Homologs (RSH). Recent studies have found that RSH can affect not only the tolerance of bacteria, but also the drug resistance and virulence of bacteria [46].

One unanticipated finding was that the virulence of the *satP* gene deletion strain was several times lower than that of the wild-type strain, and we found that the treatment rate of the mice infected with *ΔPmS* was higher. This finding broadly supports the work of other studies in this area which links tolerance with virulence [47]. However, there are also some reports contrary to this study. Ma et al. knocked out the *MazEF* gene of three strains of bacteria and found that the tolerance of the deletion strain was decreased and the virulence was enhanced, compared with the wild-type strain [48]. These findings may help us understand the relationship between drug resistance and adaptive compensation. This finding has important implications for developing inhibitors. Nevertheless, there are still many unanswered questions regarding whether *satP* can be used as a target of drug resistance inhibitors and how effective it could be in clinical application. More attention should be paid to studying *satP* and its inhibitors.

## 5. Conclusions

In this study, based on the transcriptome sequence of three strains of *Pm*, the *satP* gene was obtained, of which the expression was significantly up-regulated with the increase in drug resistance. Through the study of the formation of the *satP* gene of drug resistance, the deletion of the *satP* gene in *Pm* can lead to a faster growth rate, longer development time of drug resistance and reduced tolerance. In addition, due to the deletion of the *satP* gene, its virulence was 400 times reduced than in the wild-type strain. The results indicated that *satP* might be the target of ENR resistance inhibitors. 

## Figures and Tables

**Table 1:** QRDR mutation genotypes and fluoroquinolone MICs for *PmS*, *PmR* and *PmHR*.

Strains	MIC Range (µg/mL)				Substitution in			
	CIP	ENR	ENR + CCCP	ENR + Verapamil	GyrA	GyrB	ParC	ParE
*PmS*	0.25	0.25	0.125	0.125	-	-	-	-
*PmR*	4	4	2	2	Arg88Ile	Ser467Phe	Glu84Lys	-
*PmHR*	64	64	64	64	Arg88Ile	Ser467Phe	Glu84Lys	-

**Table 2:** QRDR mutation genotypes and fluoroquinolone MICs for *ΔPmS*, *ΔPmR* and *ΔPmHR*.

Strains	MIC Range (µg/mL)				Substitution in			
	CIP	ENR	ENR + CCCP	ENR + Verapamil	GyrA	GyrB	ParC	ParE
*ΔPmS*	0.25	0.25	0.125	0.125	-	-	-	-
*ΔPmR*	2	2	2	2	Arg88Ile	Ser467Phe	Glu84Lys	-
*ΔPmHR*	64	64	64	64	Arg88Ile	Ser467Phe	Glu84Lys	-

**Table 3:** Mutation frequency of *Pm* to ENR in six strains.

Strain	Mutation Frequency (×10^−8^) (X ± SD)
*PmS*	0.3 ± 0.1
*ΔPmS*	-
*PmR*	6.4 ± 2.8
*ΔPmR*	3.7 ± 1.5
*PmHR*	14.0 ± 5.8
*ΔPmHR*	8.2 ± 1.6

## Data Availability

The transcriptome data of the bacteria under study have been uploaded to NCBI GenBank, with the admission number 8172276. The data set generated and analyzed during this study can be obtained from the corresponding authors upon reasonable requirements.

## Supplementary Materials

The following supporting information can be downloaded at: https://www.mdpi.com/article/10.3390/vetsci10040257/s1, Figure S1. Identification of *Pm*; Figure S2. The density of FPKM; Figure S3. Histogram of differential gene between groups; Figure S4. DEGs of biological process, cellular component and molecular function GO terms; Figure S5. The enrichment bubble map of KEGG; Table S1. Primer sequence; Table S2. The quality analysis of total RNA in *Pm*; Table S3. Statistical results of trimmed reads mapping with reference genome; Table S4. The Statistics of functional annotation; Table S5. The results of top GO; Table S6. LD_50_ test results of *PmS* and *ΔPmS*.
