# Supplementary material for: Transcriptomic Changes and satP Gene Function Analysis in Pasteurella multocida with Different Levels of Resistance to Enrofloxacin
Source: Vet Sci. 2023 Mar 28;10(4):257. doi: 10.3390/vetsci10040257 (PMC10143902; doi:10.3390/vetsci10040257)
Supplement: Supplementary file 1 [file vetsci-10-00257-s001.zip › vetsci-2240257-supplementary/vetsci-2240257 Supplementary Figures.pdf]

Supplementary Figures for

Transcriptomic Changes and *satP* Gene Function Analysis in *Pasteurella multocida* with Different Levels of Resistance to Enrofloxacin

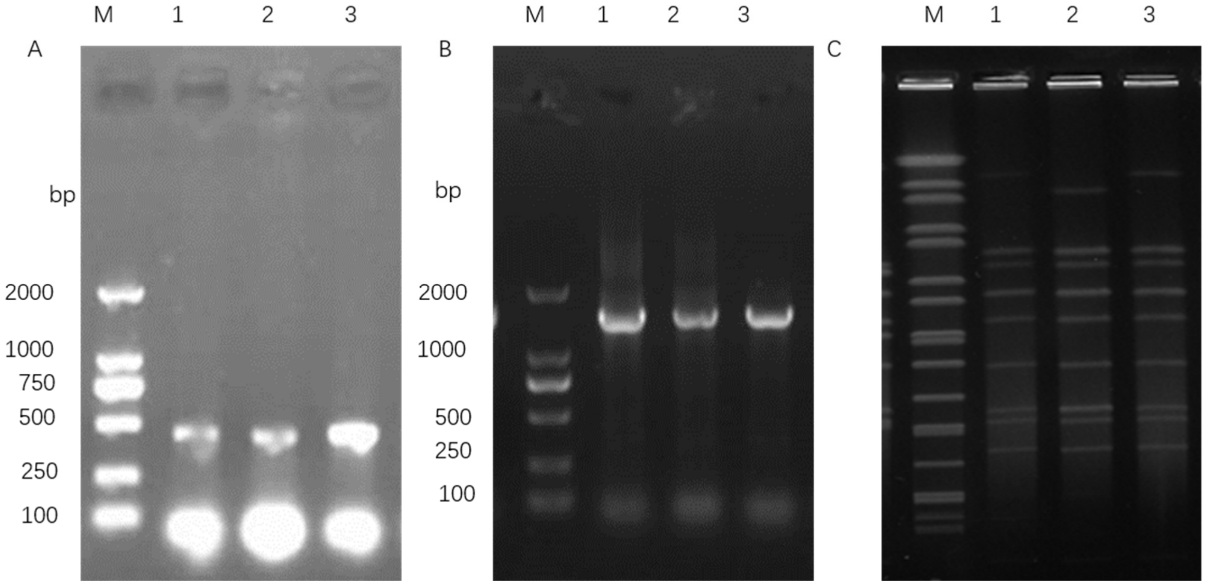

Supplementary Figure S1. Identification of *Pm* A: The PCR results of KMT1; B: The PCR results of 16S rDNA; C: The results of PFGE typing.

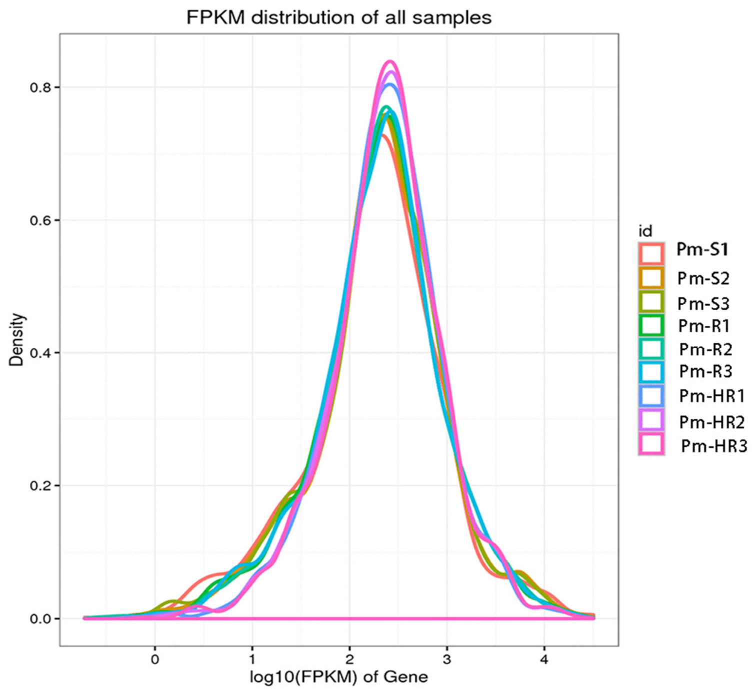

Supplementary Figure S2. The density of FPKM. The horizontal:$\log_{10}$ of FPKM; The ordinate: The density of FPKM.

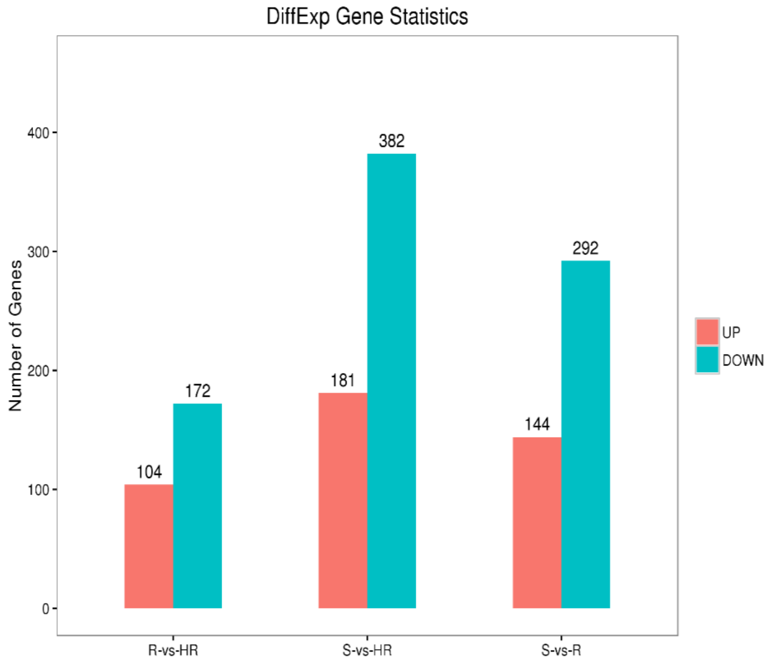

Supplementary Figure S3. Histogram of differential gene between groups. The horizontal: sample groups; The ordinate: The number of differential gene**.**

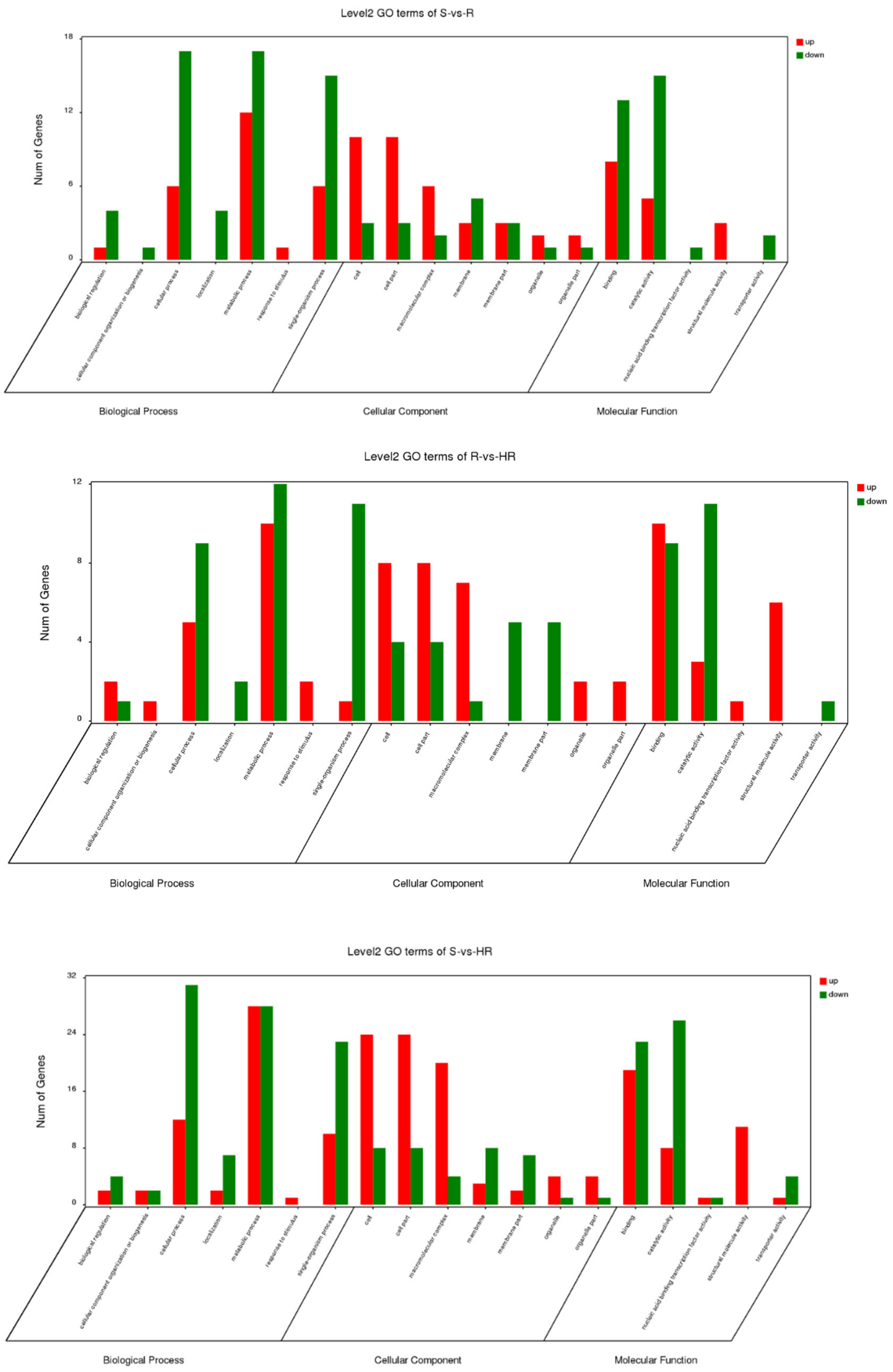

Supplementary Figure S4. DEGs of biological process, cellular component and molecular function GO terms. A: *PmS*-vs-*PmR*; B: *PmR*-vs-*PmHR*; C: *PmS*-vs-*PmHR*.

A

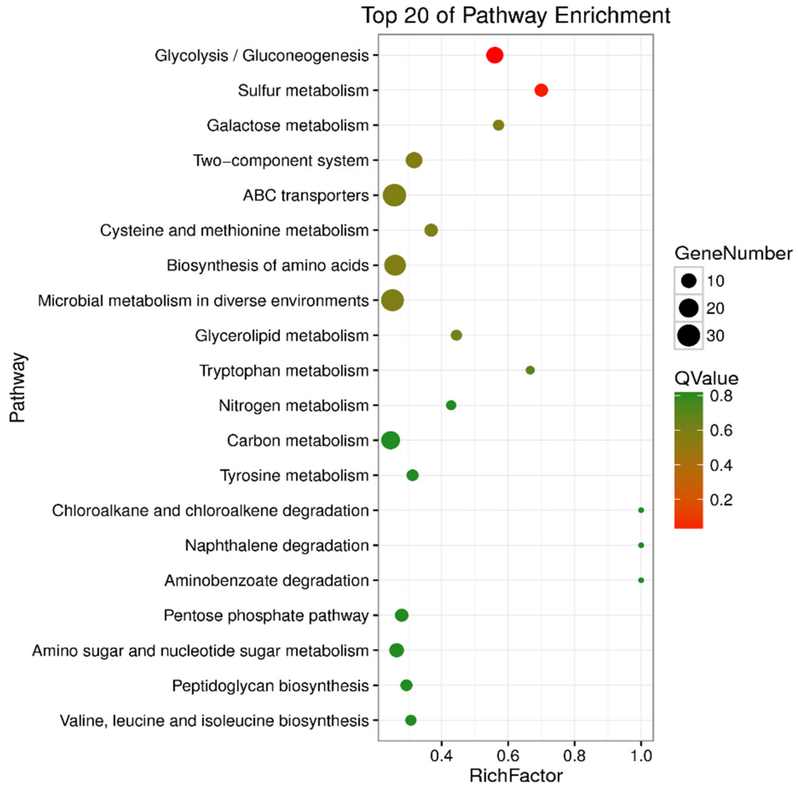

Top 20 of Pathway Enrichment
Glycolysis / Gluconeogenesis
Sulfur metabolism
Galactose metabolism
Two−component system
ABC transporters
Cysteine and methionine metabolism
Biosynthesis of amino acids
Microbial metabolism in diverse environments
Glycerolipid metabolism
Tryptophan metabolism
Nitrogen metabolism
Carbon metabolism
Tyrosine metabolism
Chloroalkane and chloroalkene degradation
Naphthalene degradation
Aminobenzoate degradation
Pentose phosphate pathway
Amino sugar and nucleotide sugar metabolism
Peptidoglycan biosynthesis
Valine, leucine and isoleucine biosynthesis
Pathway
0.4
0.6
0.8
1.0
RichFactor
GeneNumber
10
20
30
QValue
0.8
0.6
0.4
0.2

B

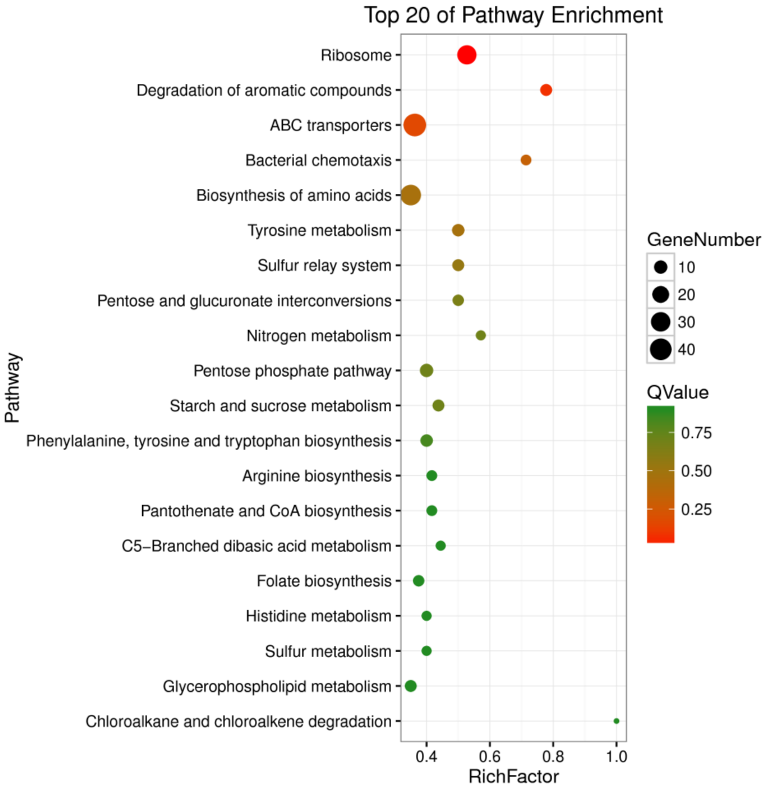

Top 20 of Pathway Enrichment
Ribosome
Degradation of aromatic compounds
ABC transporters
Bacterial chemotaxis
Biosynthesis of amino acids
Tyrosine metabolism
Sulfur relay system
Pentose and glucuronate interconversions
Nitrogen metabolism
Pentose phosphate pathway
Starch and sucrose metabolism
Phenylalanine, tyrosine and tryptophan biosynthesis
Arginine biosynthesis
Pantothenate and CoA biosynthesis
C5−Branched dibasic acid metabolism
Folate biosynthesis
Histidine metabolism
Sulfur metabolism
Glycerophospholipid metabolism
Chloroalkane and chloroalkene degradation
Pathway
0.4
0.6
0.8
1.0
RichFactor
GeneNumber
10
20
30
40
QValue
0.75
0.50
0.25

C

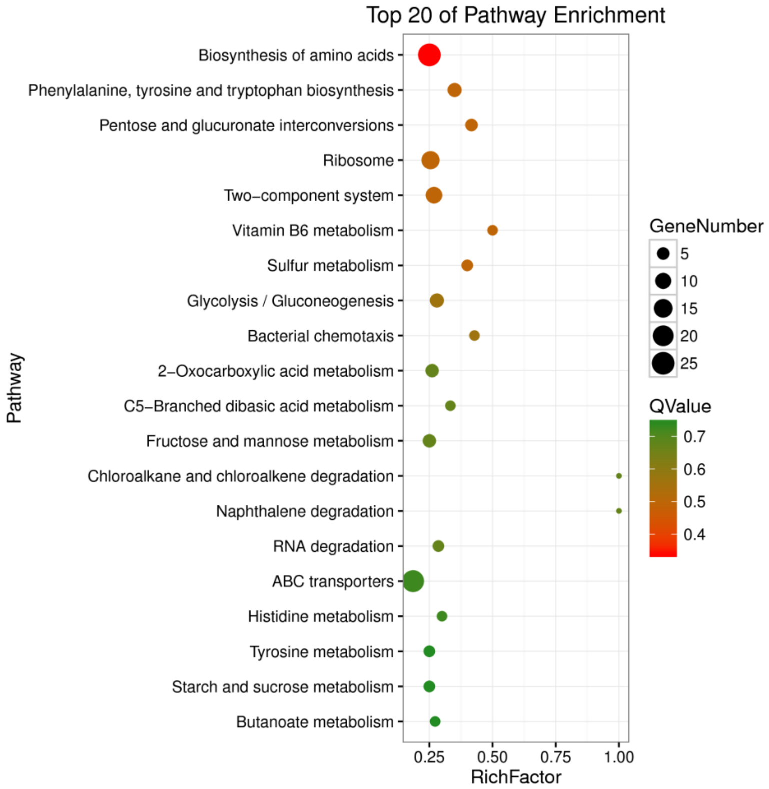

Supplementary Figure S5. The enrichment bubble map of KEGG A: *PmS*-vs-*PmR*; B: *PmR*-vs-*PmHR*; C: *PmS*-vs-*PmHR*.
