# Supplementary material for: Transcriptomic Changes and satP Gene Function Analysis in Pasteurella multocida with Different Levels of Resistance to Enrofloxacin
Source: Vet Sci. 2023 Mar 28;10(4):257. doi: 10.3390/vetsci10040257 (PMC10143902; doi:10.3390/vetsci10040257)
Supplement: Supplementary file 1 [file vetsci-10-00257-s001.zip › vetsci-2240257-supplementary/vetsci-2240257 Supplementary Tables.pdf]

Supplementary Tables for

Transcriptomic Changes and *satP* Gene Function Analysis in *Pasteurella multocida* with Different Levels of Resistance to Enrofloxacin

Supplementary Table S1. Primer sequence.

Primer name	sequence（5′→3′）
UP	F: CGTCTAGAACCATGAGCGAGATCTTTGATATT<br>R: ATCAAAAGATGGTGCGAAAAAATAAGATAAC
DOWN	F: TTTTCGCACCATCTTTTGATGATGACAT<br>R: TAGAGCTCTGTTTCATCAGCGCTGCGGTA

Supplementary Table S2. The quality analysis of total RNA in *Pm.*

Sample name	OD260/280	OD260/230	Sample name	OD260/230	OD260/230	Sample name	OD260/280	OD260/230
PMS	2.19	2.30	PMR	2.12	2.20	PMHR	2.18	1.77
PMS	2.17	2.32	PMR	2.16	2.16	PMHR	2.18	2.04
PMS	2.17	2.31	PMR	2.19	2.19	PMHR	2.20	1.98

Supplementary Table S3. Statistical results of trimmed reads mapping with reference genome.

Sample	Before Filter				After Filter			
	Clean Data(bp)	Q20 (%)	Q30 (%)	GC (%)	HQ Clean Data(bp)	Q20 (%)	Q30 (%)	GC (%)
PMS-1	2321207700	97.56	93.45	43.30	2159560579	98.44	94.65	42.69
PMS-2	1897499100	97.54	93.38	43.49	1768955797	98.39	94.52	42.87
PMS-3	1910560200	97.72	93.67	43.31	1789862837	98.46	94.68	42.76
PMR-1	2298293700	98.06	94.81	43.92	2126675340	98.92	96.01	43.32
PMR-2	2337310800	97.95	94.68	43.64	2161900037	98.90	95.97	43.04
PMR-3	2141400600	97.97	94.54	43.79	1985325044	98.82	95.71	43.15
PMHR-1	2411388900	98.09	94.70	43.17	2247057575	98.81	95.72	42.48
PMHR-2	2161450500	97.89	94.29	43.21	2013130234	98.67	95.37	42.54
PM-HR-3	1878329400	97.97	94.48	43.07	1750311733	98.74	95.54	42.40

Supplementary Table S4. The Statistics of functional annotation.

	GO	KEGG
S-vs-R	187	73
R-vs-HR	189	76
S-vs-HR	248	66

Supplementary Table S5. The results of top GO.

GO ID	FUNCTION	GO ID	FUNCTION	GO ID	FUNCTION
GO:0044425	Membrane	GO:0043229	Intracellular organelles	GO:0031252	Cellular front
GO:0031224	Inherent composition of membrane	GO:0030176	Intracellular organelles	GO:0008305	Complex integrin
GO:0098687	Chromosome region	GO:0044424	Intracellular	GO:0031301	Membrane component
GO:0031090	Organelle membrane	GO:0043228	Unbounded organelle	GO:0098636	Adhesion protein
GO:0097458	Neuronal part	GO:0043232	Intracellular organelles	GO:0016459	Myosin
GO:0098858	Cell projection	GO:0043292	Shrinkage fibre	GO:0030175	Dendrite
GO:0044427	Chromosome part	GO:0042995	Cell projection	GO:0043227	Intra-boundary organelles
GO:0044297	Cell body	GO:0005622	Intracellular	GO:0044450	Microtubule tissue
GO:0031984	Organelle region	GO:0016021	Membrane component	GO:0000781	Chromosomal telomeres
GO:0030424	Axon	GO:0043005	Neuronal projection	GO:0098796	Membrane protein
GO:0033267	Axonal part	GO:0043226	Organelle	GO:0031227	Endoplasmic reticulum part

Supplementary Table S6. LD_{50} test results of *PmS* and *ΔPmS*.

Bacteria	Number of animals /n	Dosage administered /（CFU/mL）	Dose logarithm /x	Number of dead animals /r	Mortality rate/p	Survival rate/q	pq
PmS	10	10^5	5	0	0	1	0
PmS	10	3×10^5	5.5	0	0	1	0
PmS	10	10^6	6	4	0.4	0.6	0.24
PmS	10	3×10^6	6.5	6	0.6	0.4	0.24
PmS	10	10^7	7	10	1	0	0
ΔPmS	10	10^9	9	0	0	1	0
ΔPmS	10	3×10^9	9.5	0	0	1	0
ΔPmS	10	10^{10}	10	8	0.8	0.2	0.16
ΔPmS	10	3×10^{10}	10.5	10	1	0	0
ΔPmS	10	10^{11}	11	10	1	0	0
